# Surgery

**Published:** 1893-12

**Authors:** James Swain


### SURGERY.

The treatment of over-riding fragments in oblique fractures is one that often tries all the resources of the practical surgeon, and we are indebted to Mr. Keetley[3] for the suggestion of drilling each fragment transversely to the long axis of the bone, and setting in the drill holes two steel pins (silver plated), the external ends of the pins being bent towards each other at right angles (parallel to the surface of the limb), so as to overlap, and then, while reduction is made by extension, the overlapping portions of the pins are wired together. An antiseptic dressing is applied. Senn, too, seems impressed with the defects of previous methods of securing and retaining accurate apposition in cases of compound and ununited fractures, and advocates[4] the employment of hollow perforated intra-osseous splints or of bone ferrules. He objects to the wire suture, because it frequently fails to maintain apposition; because he believes that in some cases the wire induces necrosis of the bone, and because a foreign body is thus permanently retained at the seat of fracture. Many will agree with him as to the inefficiency of the wire suture, but some of us have found that the use of a metallic nail or screw (which Senn simply dismisses with a mere mention of the methods) is often very satisfactory. The use of ivory cylinders and clamps, as recommended by Bircher[5] and Socin, have been attended by success; but they take too long to become absorbed, and spontaneous elimination is sure to take place sooner or later, for there is a limit to the absorption of aseptic absorbable bodies. The mechanical effect is the same whether a solid or hollow cylinder of ivory or bone is used, and Senn recommends the more easily absorbable intra-osseous splints of hollow perforated bone cylinders. The use of such cylinders does not interfere with the early formation of the

[3] *Lancet*, June 10, 1893, p. 1377. [4] *Annals of Surgery*, Aug., 1893.

[5] *Archiv. f. klin. Chir.*, Band xxxiv., S. 410.

intermediate callus from the medullary tissue, and the lumen of the hollow cylinder is soon filled with this valuable bone-producing material and the products of tissue proliferation. Later on new blood-vessels fill the perforations, and establish communication between the process of repair within and outside of the cylinder. Such cylinders should be made from the shafts of long bones of chickens, turkeys, or rabbits. The medullary cavity is to be increased in size by the use of a small round file, and the perforations made with a drill. The length of these intra-osseous splints will vary, according to the size of the bone and the obliquity of the fractures, from one to three inches.

In cases of very oblique fracture the bone cylinder in the cancellous tissue will not retain the fragments securely. For these cases Senn recommends the use of a bone ferrule. The rough and often denticulated fractured surfaces, held in contact by the circular splint, will bring about inter-locking of the fragments, the best safeguard against undue shortening. Angular deformity and rotation can be readily prevented by an appropriate external support. For the humerus and femur of the adult the ferrule should be prepared from the femur of an ox; for children the same bone of a smaller animal should be chosen. For the tibia the corresponding bone of the animal is chosen. With a sharp saw the shaft of the bone is cut transversely, the length of the sections varying from a quarter of an inch to an inch as desired. The medullary canal is enlarged with a round file until the thickness of the bone does not exceed one-sixth of an inch. If the ferrule is longer than an inch it should be perforated. Sterilisation is effected by boiling for an hour or more, after which the rings are kept immersed in sublimate alcohol—1 : 1000—ready for use. In applying the bone ferrule the seat of fracture must be fully exposed, and the ferrule must be large enough to slip over the fragments without danger of being broken. The ferrule is slipped over the most accessible fragment, and after reduction is effected is passed along so as to engage the second fragment. A trustworthy assistant should hold the limb in proper position, as bending at the seat of fracture might break the ring.

In the use of the cylinder or ferrule immobility at the seat of fracture is maintained by the application of a plaster-of-Paris splint to the limb, and this can be fenestrated if the wound has been drained.

The treatment of fistula-in-ano by excision and suture often diminishes the period of convalescence by some weeks, and is the operation which should usually be adopted; for even if it does not entirely succeed in effecting union by first intention, the patient is no worse off for the attempt to obtain that result. The technique of this method as described by Thévenard,[1] who

[1] *Arch. gén. de Méd.*, April, 1893, quoted in *Med. Chron.*, Sept., 1893, p. 408.

considers it good in all cases except those where the burrowing is very extensive, is as follows: Rigid asepticity of the field of operation, which is absolutely essential, is obtained by the daily administration of a saline purgative and a gramme of naphthol during the preceding week, and on the day before operation these drugs are replaced by a dose of 5—10 centigrammes of opium, and a boracic acid injection is given morning and evening, and again the next morning, to ensure emptiness of the rectum. The ano-perineal region is carefully shaved and cleansed. The patient being anæsthetised, and placed in the lithotomy position, the surgeon proceeds to excise the fistulous track. "If it is a single one, a grooved sound is passed by the cutaneous orifice, emerging into the rectum at the other end. An incision is then made into the mucosa down to the indurated tissues bounding the sinus. With strong forceps these tissues are fixed on the sound, and then dissected from above downwards, the sound and the sinus being removed together. The cutaneous scar is then freshened, and then the sutures are passed. In the same manner the secondary burrows and diverticula are sought for and laid open in their turn. The excision of the diseased tissues is then proceeded with." The curette must be used instead of the bistoury if the burrowing is very deep. Thévenard uses interrupted stitches of catgut placed in order from above down; but I have generally found a continuous catgut stitch passed with a fully-curved needle more manageable in the deeper parts, while the skin surface is brought together by interrupted sutures of silk-worm gut. Iodoform is then dusted on the parts, and a pad of wool applied by means of a T bandage. The bowels may be relieved by an enema of glycerine about the fourth or fifth day, the skin sutures being removed about the seventh to tenth day. Thévenard gives particulars of thirteen cases of different degrees of complexity, and in all the operation was successful, although several of them were tubercular. The advantages of this method of treatment over the usual plan of slitting up the fistula and allowing the wound to granulate are: less risk of secondary infection of the wound from the rectal secretions, &c.; shorter time (four or five weeks only for most complicated cases) required for cure; and the functional integrity of the anal sphincter is absolutely maintained, for when this has been incised its muscular fibres are taken up by the deep sutures and the ring restored to its normal condition.

"To sum up, the method of suture is not alone applicable in simple cases, but is specially indicated in complicated fistulæ, and in tuberculous subjects is essential."

Dr. Howard complains[1] of the unsatisfactory results and prolonged treatment necessary in cases of in-growing toe-nail

[1] *N.Y. Med. Journ.*, May 27, 1893, p. 579.

which have resisted ordinary methods, and demand some such treatment as avulsion of the nail, with or without destruction of the matrix. The new nail after avulsion is often thickened and deformed, and gives as much trouble as before; and a similar failure may attend the destruction of the matrix, which not infrequently leaves a few hard nodules which become the cause of much pain. The operation for this persistent affection proposed by Dr. Howard is as follows: Commencing about three-sixteenths of an inch from the edge of the nail, passing the knife directly towards the bone (not going deep enough to wound the periosteum), make the cut from the centre in front, horizontal to the plantar surface, around and back to a line a little beyond the proximal end of the nail; next begin at the same place as before, pass the knife in a semi-circular manner, ending with the proximal end of the first cut, removing an elliptical wedge-shaped section by bringing the cuts together at their deepest angle. A piece three-eighths of an inch in width is often sufficient to draw the soft parts away from the nail when closed. Bring the edges together with deep silk-worm gut sutures, and dress antiseptically. In from a week to ten days a shoe may be worn with perfect comfort. The operation may be varied to suit the case; for instance, where both corners are ingrown, or where the nail has been removed and is short and sharply incurved, and allows the end of the toe to rise all around above the nail, the cut should be made around the entire end of the toe, and a piece sufficiently large removed to bring the nail above the surrounding soft parts.

Dr. Howard has operated by this method in twenty cases, and all have been successful. I have recently tried this operation in three cases of medium severity, supplementing the steps described above by slicing away the exuberant granulations at the side of the nail. In each case union by first intention occurred; the stitches were removed in about a week; and, so far as one can judge from so limited a number of cases, the final result was more satisfactory, and achieved more quickly than by ordinary operative procedures.

Conservative operations on the ovaries, tubes, and uterus have been practised now for some years, and seem to meet with a good measure of success.

Mr. A. C. Butler-Smythe has stated[1] that where the operator finds that the ovaries and tubes are only partially adherent, and where no visible or tangible disease can be discovered in these organs, he regards their removal as unnecessary, and believes that by breaking down the adhesions (upon which the symptoms of pain, &c., depend) the patient can

[1] *Lancet*, Feb. 21, 1891, p. 424.

be cured. He says: "I have now operated on six occasions for adherent tubes and ovaries, and in every instance have been successful in relieving the appendages and benefiting the patients. I have also assisted at four operations of a similar nature where the results were favourable beyond expectation." [1]

These conservative operations have been extensively performed by A. Martin, and he draws attention [2] to the importance of this subject.

I. *Resection of the ovary.*—Ovarian disease is very frequently bilateral, but sometimes circumscribed cystic disease may be found in the other ovary. The question arises as to whether the whole organ should then be removed. It certainly should be if there is no healthy ovarian tissue left, or the process be a suppurative one; but in some other cases it may not be necessary to remove it. He refers to twenty-seven such cases with one death; two of these relapsed, and of the twenty-four remaining ones eight bore children. Ignipuncture has been employed, but the author is satisfied with incision and stitching up.

II. *Resection of a stenosed tube, the other being removed for disease.*—Here it may be more difficult to recognise the character of the disease. The contents of the tube must be most carefully looked to. If the contents be turbid or unmistakably purulent, or if the mucous membrane be ulcerated, the tube must be removed, otherwise resection with the formation of a new ostium may be practised. If any doubt exists, the whole appendage must be taken away. Of forty cases with two deaths, only four were not cured or considerably improved. Only one became pregnant, but twelve were unmarried, and the husbands of some others were neurasthenical or had had gonorrhœa.

III. *Enucleation of myomata.*—The older the patient the more likely are myomata to be multipled. In one hundred and forty-one cases of intra-parietal myomata twenty-six died, but this includes the period of development of the technique of laparotomy. Of the last twenty, only one died. Of the one hundred and fifteen, only four relapsed (3 per cent.). The author has not found any difficulty in stitching up the bed of the tumour, nor has unpleasant hemorrhage occurred. Only two of these patients became pregnant, but other causes of sterility may exist.

The author concludes: (i.) that the conservative operations do not present any materially greater risk than radical ones; (ii.) that women are thus relieved of their troubles in by far the

1 *Loc. cit.*

2 *Deut. med. Woch.*, July 27, 1893, quoted in *Brit. Med. Journ.*, Sept. 9, 1893.

greater majority of cases; (iii.) that relapses are exceptional; (iv.) that the female functions persist; (v.) that child-bearing is possible; (vi.) that child-birth then takes place without any special risk.

JAMES SWAIN.

